# Resistance profiles of urinary tract infections in general practice - an observational study

**DOI:** 10.1186/1471-2490-12-33

**Published:** 2012-11-21

**Authors:** Guido Schmiemann, Ildikó Gágyor, Eva Hummers-Pradier, Jutta Bleidorn

**Affiliations:** 1 Department for Health Services Research, Institute for Public Health and Nursing Science, Universität Bremen, Bremen, Germany; 2 Department of General Practice and Family Medicine, University of Goettingen, Göttingen, Germany; 3 Institute of General Practice and Family Medicine, Hanover Medical School, Hannover, Germany

**Keywords:** Urinary tract infection, Primary care, Drug resistance, Anti-bacterial agents

## Abstract

**Background:**

Guideline recommendations on therapy in urinary tract infections are based on antibiotic resistance rates. Due to a lack of surveillance data, little is known about resistance rates in uncomplicated urinary tract infection (UTI) in general practice in Germany. In a prospective observational study, urine cultures of all women presenting with urinary tract infections in general practice were analysed. Resistance rates against antibiotics recommended in German guidelines on UTI are presented.

**Methods:**

In a prospective, multi-center observational study general practitioner included all female patients ≥ 18 years with clinically suspected urinary tract infection. Only patients receiving an antibiotic therapy within the last two weeks were excluded.

**Results:**

40 practices recruited 191 female patients (mean age 52 years; range 18–96) with urinary tract infections. Main causative agent was Escherichia coli (79%) followed by Enterococcus faecalis (14%) and Klebsiella pneumoniae (7.3%).

Susceptibiliy of E.coli as the main causative agent was highest against fosfomycin and nitrofurantoin, with low resistance rates of 4,5%; 2,2%. In 17,5%, E.coli was resistant to trimethoprim and in 8,5% to ciprofloxacin.

**Conclusions:**

Resistance rates of uropathogens from unselected patients in general practice differ from routinely collected laboratory data. These results can have an impact on antibiotic prescribing and treatment recommendations.

## Background

Antibiotic resistance is an emerging and serious public health problem resulting in increased morbidity and mortality. In urinary tract infections (UTI), resistance rates against commonly prescribed antimicrobial agents are constantly rising. Nowadays, in many countries more than 20% of responsible uropathogens are resistant to trimethoprim /sulfamethoxazole (tmp-smx) and to cephalosporins. This increasing resistance is also being observed for fluoroquinolones with resistance rates, risen up to 10% [1,2]. As medicine faces a bleak outlook on availability of effective antibiotic treatment, new therapeutic strategies for urinary tract infections are necessary.

In 2011 an action plan has been launched by the European Commission [3] to tackle these problems. Aims and strategies include the promotion of a restrictive and appropriate use of antibiotics as well as the promotion of national surveillance programs. To optimize antibiotic treatment of uncomplicated UTI, the latter is urgently required for several reasons:

● Although the majority of antibiotic prescribing for uncomplicated UTI takes place in primary care, information on antibiotic resistance is mainly based on data from hospitals or laboratories, i.e. - highly selected patients. Thus, results cannot be generalized to UTI patients in general practice who are likely to present with lower resistance rates.

● In general practice, urine sample of patients with UTI are not routinely tested for resistance patterns. According to German guidelines on UTI [4,5], resistance testing is recommended only in cases of treatment failure or suspected complications.

● Resistance patterns of causative uropathogens are known to vary considerably between regions and countries [1].

In Germany, as in many other countries, there is no sentinel network which routinely assesses resistance rates of urinary tract infections in general practice [6].

In face of all these facts, a prospective observational study to target antibiotic therapy in uncomplicated UTI tackles the following questions:

Which uropathogens cause uncomplicated UTI in general practice?

To what extent are these uropathogens susceptible to antibiotics recommended in German guidelines?

## Method

The study idea emerged in a web based discussion forum of German general practitioners hosted by the German College of General Practitioners and Family Physicians. A prospective multi-center observational study design was set up. 579 physicians participating in the web based discussion forum and 193 teaching practices of the Institute of General Practice of Hannover Medical School were invited for participation via e-mail.

During a six week period in autumn 2011, participating practices were asked to include all female patients ≥ 18 years in whom urinary tract infection was suspected. The only exclusion criterion was antibiotic therapy within the last two weeks.

After written consent was obtained, patients were asked to provide a clean catch midstream urine sample. The sample was sent to the local laboratory. All laboratories were asked to perform resistance testing at least for trimethoprim, ciprofloxacin, nitrofurantoin and fosfomycin, even if these antibiotics were not being tested routinely.

For classification of UTI and prevailing risk factors, patient data on age, pregnancy status, indwelling urine catheter, risk factors for a complicated infection (i.e. hospital stay within the last 2 weeks, immunosuppression, or neurological disease affecting micturition), and previous UTI were documented by the general practitioners (GP) from patients’ records.

Patient data and laboratory results were entered into a web based survey instrument (lime-survey®) in anonymous form. Access to the database was restricted by a personal access token and performed by medical staff. The patients themselves had no access to the database.

For financial reasons – the study was conducted without external funding - a central laboratory was not feasible. Laboratories conducting urine analyses have to be certified and have to use standardized methods, either DIN, EuCast or CLSI. All urine cultures/susceptibility tests were performed in the laboratories the practices were attached to. Susceptibility of the causative bacteria was categorized as susceptible (S), intermediate (I) or resistant (R).

Quality control: a randomized sample of 5% of all patients (n = 14) was drawn by the investigators. A study assistant compared patient data and results of susceptibility testing entered in the web based survey with the original data held on file in the practice. The comparison was based on a re- identification code known to the practice only. Neither causative agents nor susceptibility results had to be corrected due to the quality control.

Descriptive data analysis (absolute and relative frequencies) was performed descriptively using Microsoft Excel^©^.

Microbiological methods: Native midstream urine was sent to the laboratories. Laboratories were asked to perform a routine urine culture and identification of pathogens as well as susceptibility testing including trimethoprim, fosfomycine-trometamol, nitrofurantoin and ciprofloxacin. These agents were chosen as they are recommended by national guidelines [5]. Positive urine culture was defined as bacterial count ≥ 10^3^ cfu/ml.

Approval of the ethics committee of Hannover Medical School was obtained (N0. 1138 2011).

## Results

### Participating practices/patient recruitment

67 practices expressed their willingness to participate, 40 practices included patients (Ø 4.8 patients/practice). Patient recruitment was not possible for some practices (n = 4) as their laboratory would not provide the recommended antibiotic tests against trimethoprim or fosfomycin. [Most of the participating practices were from Lower Saxony (28/40).]

Within the six week study period, most practices (n = 25) included 1–5 patients; 13 practices included 6–10 patients and two practices included more than 11 patients.

### Patient characteristics

191 women with positive urine culture were included. The mean age was 51.6 years (SD 21.7; Range 18–96). The most prevalent risk factors for UTI were diabetes mellitus (9.6%; 18/191) and recurrent UTI 18.3% (n = 35). (see Table 1 for details).

**Table 1:** Patient characteristics

	**% Patients (n = 191)**
Age	51.6 (SD 21.7, Range 18–96)
Diabetes mellitus	9.6%
Recurrent UTI	18.3%
Pregnant	0%
Indwelling urinary catheter	0%
Other risk factor	3.1%

Characteristics of 191 female patients with urinary tract infections. SD = standard deviation, Recurrent UTI = Previous Urinary tract infection in the last 6 month; Other risk factors = hospital stay within the last 2 weeks, immunosuppression or neurological disease affecting micturition.

### Urine culture and uropathogens

In 36.1% of urine cultures, more than one species could be identified; relevant uropathogens from all cultures were included.

Escherichia coli was found to be the causative pathogen in 72.8% (139/191). Other typical uropathogens found were Enterococcus faecalis (n = 26; 13.6%), Klebsiella pneumoniae (n = 14; 7.3%), Proteus mirabilis (n = 11) (see Table 2).

**Table 2:** Bacteria detected and susceptibility profile

**Isolates**	**n (%)**	**Trimethoprim**			**Fosfomycin**			**Nitrofurantoin**			**Ciprofloxacin**		
		**S n (%)**	**I n (%)**	**R n (%)**	**S n (%)**	**I n (%)**	**R n (%)**	**S n (%)**	**I n (%)**	**R n (%)**	**S n (%)**	**I n (%)**	**R n (%)**
All	191 (100)												
E. coli	139 (72.7)	105 (80.8)	2 (1.7)	23 (17.5)	126 (95.5)	0 (0)	6 (4.5)	130 (94.2)	5 (3.6)	3 (2.2)	126 (91.3)	0 (0)	12 (8.7)
Other typical uropathogens	71 (37.2)	28 (50)	0	28 (50)	46 (76,8)	1 (1.7)	13 (21.7)	43 (68.2)	5 (7.9)	15 (23.8)	48 (77.4)	9 (14.5)	5 (8.1)
Enterococcus faecalis	26 (13.6)	6	0	14	15	1	6	21	0	2	16	5	2
Proteus mirabilis	11 (5.7)	1	0	8	8	0	0	0	0	10	9	0	0
Staph. saprophyticus	4 (2.1)	4	0	0	3	0	1	4	0	0	4	0	0
Staph. others	5 (2.6)	1	0	2	3	0	0	1	2	0	2	1	0
Strept. agalacticae	8 (4.2)	2	0	3	7	0	0	7	0	0	2	3	2
Kleb pneumoniae	14 (7.3)	12	0	1	10	0	4	9	2	3	13	0	1
Staph. aureus	3 (1.6)	2	0	0	0	0	2	1	1	0	2	0	0

In the first column typical uropathogens and the number of isolates are documented. In the upper two rows results of susceptibility testing against E. coli and all other typical uropathogens are presented. Due to the low numbers no percent rates are given in the remaining rows.

### Susceptibility testing

Susceptibiliy of E.coli as the main causative agent was highest against fosfomycin and nitrofurantoin, with low resistance rates of 4,5% resp. 2,2%. In 17,5%, E.coli was resistant to TMP and in 8,5% to ciprofloxacin. In contrast to E.coli, other typical uropathogens showed higher resistance rates for TMP, fosfomycin and nitrofurantoin (50%, 21.7%, 23.8%).

Ciprofloxacin is equally effective against E. coli and other typical uropathogens (8.7%; 8.1%). Further results on susceptibility are presented in detail in Table 2.

In a few cases a urine culture was not tested for all antibiotics requested. This refers mainly to trimethoprim (130/139 samples were tested) and reflects the lack of a standard in antibiotic testing among laboratories (see Table 2).

### Recurrent UTI

35 patients had experienced a previous urinary tract infection in the last six months. Besides E.coli (29/35), causative pathogens in recurrent infections were Staphylococcus aureus, Enterococcus faecalis, Proteus mirabilis, Staphylococcus saprophyticus and Klebsiella pneumoniae.

In recurrent UTI, E.coli showed higher resistance rates against trimethoprim (25%) and ciprofloxacin (17%) while resistance rates for nitrofurantoin (3.4%) and fosfomycin (0%) were very low.

## Discussion

In this prospective observational study, causative agents and susceptibility results in uncomplicated UTI in general practice were collected with a pragmatic approach. Though participating practices constitute a (self-selected) convenience sample which cannot be considered representative, practices were located widely across (predominately north western) Germany. Pragmatic inclusion criteria were given for patients to facilitate recruitment, based on the GPs clinical judgment, which is usually based on patient history and symptoms. While participating practices are likely to be more interested in research (and possibly in antibiotic resistance) than non-participating or non-recruiting practices, it seems unlikely that their patients are different [7].

Thus, general practice based information on resistance patterns of typical uropathogens in general practice in Germany can be provided for the first time. Comparing our results with reports of routinely collected data from a more specialized level of care [8,9] revealed relevant differences (Table 3). Antibiotic resistance against trimethoprim, a drug recommended by many guidelines on urinary tract infections [5,10,11] is obviously lower in general practice patients compared with laboratory surveillance data. Ciprofloxacin, an antibiotic recommended for complicated infections, also shows higher susceptibility in primary care. Fosfomycin and nitrofurantoin still have remarkably low resistance rates, probably due to quite low prescription rates in Germany. Our results confirm that urine cultures submitted to laboratories do not represent the susceptibility patterns in unselected patients [12,13]since in these patients urine cultures are only requested if the patient fails to respond to treatment, has recurrent episodes or other complicating factors. Thus, in the primary care setting, urine cultures for suspected UTI remain exceptional and represent complicated rather than typical uncomplicated patients. Therefore, data from laboratory results [8,9] or studies including data from patients in specialist care (urology, gynecology) [14] differ from our results, they do not represent the antimicrobial resistance situation in general practice in Germany.

**Table 3:** Resistance rates in different settings

	**Our results**	**Robert Koch Institute[**[7]**]**	**Antibiotic resistance monitoring in Lower Saxony[**[8]**]**	**ARESC[**[13]**]**
TMP	17.5	29.2	28.7	25.9
Nitrofurantoin	2.2	n.a	1.2	4.5
Fosfomycin	4.5	n.a	n.a	0.8
Ciprofloxacin	8.7	18.2	13.9	4.5

With exception of our study all others used TMP-SMX. N.A = No information on susceptibility rates given.

The strength of our study is its pragmatic approach in the general practice setting, resulting in a high number of (uncomplicated) patients recruited in a very short period of time. A direct comparison with two epidemiological German studies [14,15] is limited as these studies included only women up to 65 years under specialists care. This may well explain the low number of patients with diabetes mellitus (4.8%) found by Wagenlehner compared with 9.6% in our study including elderly women. The lack of a central laboratory resulting in different sets of antibiotics used for routine susceptibility testing, or available for testing at all proved a limit in obtaining comparable susceptibility data for all patients within our study. However, pooling results from different certified laboratories is routinely done when reporting of antimicrobial resistance rates [8]. Therefore the fact of using pooled data from different local laboratories instead of a central one is unlikely to bias our results. In any case, it reflects usual practice and usual community based care in Germany.

National or regional standards for the selection of antibiotics to be included in susceptibility analysis are missing in Germany, as well as legal pressure to include antibiotics recommended in guidelines. In many microbiological laboratories, routinely conducted tests often do not include all antimicrobial agents recommended by current guidelines, for example, fosfomycin-trometamol or nitrofurantoin are often missing. This constitutes a very relevant barrier against guideline implementation and appropriate use of antibiotics.

Resistance rates of common uropathogens have an important impact on guideline recommendations regarding the choice of antibiotics. Although there is no linear correlation between resistance level and strength of a recommendation, rising resistance rates compromise acceptance of a recommendation, and confirmed lower resistance profiles imply the need to review a guideline recommendation. Based on expert consensus, usually a resistance level of >20% is used as a cut-off in guidelines on urinary tract infections [16,17].

With a resistance rate of 17.5% for the main causative agent E. coli, our results confirm the recommendation of TMP as a first choice antibiotic substance in the German primary care setting.

An urgent task for the future is to build up a sentinel network. Only by collecting resistance data permanently, a tailored and specific antibiotic therapy of UTI is permitted. This also implies implementation of guideline-adjusted resistance testing in medical laboratories.

## Conclusions

Resistance rates of uropathogens from unselected patients in general practice differ from routinely collected data from laboratories. These results have a major effect on antibiotic prescribing and treatment recommendations. Sentinel networks for a representative UTI resistance data are urgently needed to monitor UTI resistances in general practice.

## Competing interests

All authors declare that they have no competing interests.

## Authors’ contributions

GS and JB conceived the design of the study with contribution from IG and EHP. GS drafted the manuscript and JB, IG and EHP contributed to the manuscript. All authors read and approved the final manuscript.

## Pre-publication history

The pre-publication history for this paper can be accessed here:

http://www.biomedcentral.com/1471-2490/12/33/prepub

## Achnowledgements

We gratefully thank all participating practices. Thanks to Daniel Dettmer for his support in data management.
